# Structure of cyclin G-associated kinase (GAK) trapped in different conformations using nanobodies

**DOI:** 10.1042/BJ20131399

**Published:** 2014-03-14

**Authors:** Apirat Chaikuad, Tracy Keates, Cécile Vincke, Melanie Kaufholz, Michael Zenn, Bastian Zimmermann, Carlos Gutiérrez, Rong-guang Zhang, Catherine Hatzos-Skintges, Andrzej Joachimiak, Serge Muyldermans, Friedrich W. Herberg, Stefan Knapp, Susanne Müller

**Affiliations:** *University of Oxford, Target Discovery Institute (TDI) and Structural Genomics Consortium (SGC), Old Road Campus Research Building, Oxford OX3 7DQ, U.K.; †Research Unit of Cellular and Molecular Immunology and Department of Structural Biology, VIB, Vrije Universiteit Brussel, Pleinlaan 2, 1050 Brussels, Belgium; ‡Department of Biochemistry, University of Kassel, Heinrich-Plett Strasse 40, 34132 Kassel, Germany; §Biaffin GmbH & CoKG, Heinrich-Plett Strasse 40, 34132 Kassel, Germany; ¶Department of Animal Medicine and Surgery, Veterinary Faculty, University of Las Palmas de Gran Canaria, 35416, Arucas, Las Palmas, Spain; ∥Midwest Center for Structural Genomics and Structural Biology Center, Biosciences Division, Argonne National Laboratory, 9700 South Cass Avenue, Argonne, IL 60439, U.S.A.

**Keywords:** activation loop, cyclin G-associated kinase, drug side effect, kinase inhibitor, nanobody, protein structure, ASCH, activation segment C-terminal helix, AUC, analytical ultracentrifugation, CDR, complementarity-determining region, DARPin, designed ankyrin-repeat protein, EGFR, epidermal growth factor receptor, GAK, cyclin G-associated kinase, HA, haemagglutinin, MPSK1, myristoylated and palmitoylated serine/threonine kinase 1, NAK, numb-associated kinase, Nb, nanobody, RU, resonance unit, SeMet, selenomethionine, SPR, surface plasmon resonance, TCEP, tris-(2-carboxyethyl)phosphine, TEV, Tobacco etch virus

## Abstract

GAK (cyclin G-associated kinase) is a key regulator of clathrin-coated vesicle trafficking and plays a central role during development. Additionally, due to the unusually high plasticity of its catalytic domain, it is a frequent ‘off-target’ of clinical kinase inhibitors associated with respiratory side effects of these drugs. In the present paper, we determined the crystal structure of the GAK catalytic domain alone and in complex with specific single-chain antibodies (nanobodies). GAK is constitutively active and weakly associates in solution. The GAK apo structure revealed a dimeric inactive state of the catalytic domain mediated by an unusual activation segment interaction. Co-crystallization with the nanobody NbGAK_4 trapped GAK in a dimeric arrangement similar to the one observed in the apo structure, whereas NbGAK_1 captured the activation segment of monomeric GAK in a well-ordered conformation, representing features of the active kinase. The presented structural and biochemical data provide insight into the domain plasticity of GAK and demonstrate the utility of nanobodies to gain insight into conformational changes of dynamic molecules. In addition, we present structural data on the binding mode of ATP mimetic inhibitors and enzyme kinetic data, which will support rational inhibitor design of inhibitors to reduce the off-target effect on GAK.

## Abstract 2

Cyclin G-associated kinase (GAK) is a regulator of clathrin-coated vesicle trafficking. The determined crystal structures of GAK in complex with specific single chain antibodies (nanobodies) revealed the domain plasticity of this kinase and unusual activation segment architecture.

## INTRODUCTION

Kinases are highly dynamic enzymes and key regulators of signalling pathways. The proper timely and spatial activation of these pathways requires fine tuning and a tight regulation of the proteins involved. In a crystal structure only one conformation of a kinase is trapped and stabilized by crystal contacts. Understanding the function of a kinase requires understanding of its structural plasticity and, as a consequence, the generation of multiple structures in diverse conformations.

GAK (cyclin G-associated kinase) is a member of the NAK (numb-associated kinase) family, which in humans also includes STK16 (serine/threonine kinase 16)/MPSK1 (myristoylated and palmitoylated serine/threonine kinase 1), AAK1 (adaptor-associated kinase 1) and BIKE [BMP2 (bone morphogenetic protein 2)-inducible kinase]. This small subfamily of kinases is located in the centre of the kinase phylogenetic tree and shows a large sequence and structure diversity to other kinases as well as within the NAK subfamily [1]. GAK was originally identified as an association partner of cyclin G and CDK5 (cyclin-dependent kinase 5) [2]. It is essential for clathrin trafficking, mediating binding of clathrin to the plasma membrane and the *trans*-Golgi network, and regulating receptor signalling by influencing trafficking downstream of clathrin-coated vesicles [3,4]. In addition to its role in the cytoplasm, GAK has important functions in the nucleus. It is required for the maintenance of proper centrosome maturation and progression through mitosis [5]. GAK plays a central role during development and GAK^−/−^ mice die early in gestation. Deleting GAK in adult mice in an inducible knockout mouse model resulted in death of these animals after 3 weeks, demonstrating that GAK is also essential for the viability of adult mice. Tissue-specific conditional knockout of GAK in the skin, liver or developing brain also resulted in lethal phenotypes shortly after birth with severe alteration of the affected tissues and a failure of progenitor cells to differentiate [6]. The kinase activity of GAK plays an essential role in viability and mice expressing only a kinase-dead form of GAK also die shortly after birth. Death is caused by respiratory dysfunction due to altered distribution of surfactant protein [7]. This critical role for GAK in the maintenance of respiratory function is thought to be the cause of the side effects observed with the EGFR (epidermal growth factor receptor) kinase inhibitor gefitinib, used in the treatment of patients with NSCLC (non-small-cell lung cancer), as GAK is potently inhibited by this drug [7].

Activation of kinases frequently occurs via phosphorylation of the activation loop, also called the T-loop, a stretch of usually 30–40 amino acids that is located between two conserved motifs found in kinases: the DFG (Asp/Phe/Gly) and APE (Ala/Pro/Glu) motifs. Of the NAK family of kinases only the structure of MPSK1 has been determined so far. This structure is characterized by an unusual activation segment comprising an additional β-sheet and a large α-helical insertion, termed the ASCH (activation segment C-terminal helix), which is anchored to the lower kinase lobe by a number of family-specific hydrophobic interactions [8]. This unusual arrangement has been predicted to be present in all NAK family members, but the presence of this atypical activation segment has not been confirmed experimentally.

Nbs (nanobodies) are robust small (15 kDa) single domain antigen-binding fragments derived from heavy chain-only antibodies, naturally occurring in Camelids, which can efficiently be produced by a variety of different hosts [9,10]. They contain a more diverse structural repertoire of their paratopes than mouse or human antibodies, as their hypervariable loops can adopt new folds not observed in conventional mouse or human antibodies. Their antigen-recognition sites typically form a convex surface with a large CDR (complementarity-determining region) loop 3 (CDR3) capable of reaching antigen-binding pockets that are less antigenic for other types of binders. It is thought that this helps to compensate for the loss of repertoire caused by the loss of the combinatorial VH-VL diversity [11]. In addition, high solubility due to specific hydrophilic amino acids found in the framework-two regions between CDRs, as well as their resistance to temperature and denaturing, make them ideally suitable for application in structural biology [12,13]. High-affinity Nbs and other affinity reagents are thought to be conformation sensitive [14,15], thus using them in crystallization complexes can aid in the determination of conformation-specific structures of dynamic molecules such as kinases.

In the present paper, we report the first crystal structures of the GAK catalytic domain. The apo structure of GAK revealed a dimeric assembly mediated by the disordered elongated activation segment that dislodges from the lower lobe to interact with a second kinase molecule. Weak association of the GAK kinase domain was also observed in solution using AUC (analytical ultracentrifugation). Additional GAK and Nb co-crystalization structures revealed two distinct kinase conformations: one capturing a conformation similar to the one observed in the unliganded GAK kinase domain confirming the dimeric arrangement in a different crystal form, and a second structure with an active conformation of GAK revealing a folded activation segment architecture characterized by the insertion of an unusual β-sheet and the ASCH that has also been described in MPSK1, suggesting a conserved architecture of the activation segment in NAKs. The different conformations observed in these three diverse crystal structures revealed a large degree of domain plasticity that include significant changes in the GAK active site providing a rational for the potent inhibition of GAK by a large diversity of kinase inhibitors.

## EXPERIMENTAL

### Cloning and protein purification

DNA fragments encoding the kinase domain of human GAK (residues 14–351 for the co-crystal structure or 12–347 for the apo structure) were subcloned into either the pNIC-H102 or pNIC28-Bsa4 vector, both incorporating an N-terminal TEV (tobacco etch virus)-cleavable His_6_ tag. The recombinant protein was expressed in *Escherichia coli* BL21(DE3)-R3 cells cultured in LB medium at 37°C and induced with 0.5 mM IPTG at 18°C overnight. For the SeMet (selenomethionine)-labelled protein, 90 mg of SeMet and 150 mg each of inhibitory amino acids (VILKTF) was added to the cultures as described previously [16]. Cells were harvested and resuspended in lysis buffer comprising 50 mM Hepes (pH 7.5), 500 mM NaCl, 5 mM imidazole, 5% glycerol and 0.5 mM TCEP [tris-(2-carboxyethyl)phosphine]. After breaking the cells by sonication, the supernatant was separated by centrifugation (55914 ***g*** for 60 min at 4°C) and the proteins were purified by Ni-affinity and size-exclusion (Superdex S200) chromatography. The His_6_ tag was removed by TEV protease treatment, after which the cleaved protein was passed over Ni–Sepharose resin. The pure protein was stored in storage buffer [10 mM Hepes (pH 7.5), 300 mM NaCl, 5% glycerol and 0.5 mM TCEP] at −80°C.

### Nb generation and purification

A dromedary (Veterinary Faculty, University of Las Palmas, Spain) was immunized using injections of 100 μg of GAK protein in adjuvant. Blood was collected 4 days after the last boost injection. Library generation, phage display, Nb expression and purification were performed according to procedures described in [17]. All animal vaccination experiments were performed in strict accordance with good practices, following EU animal welfare legislation. Every effort was made to minimize suffering. Briefly, after subcloning the variable domain repertoire in the pMECS phage display vector, which adds an HA (haemagglutinin) and a His tag, a library of 1.2×10^7^ transformants, which has been panned on recombinant GAK, was generated, of which 78% had correctly sized inserts. The Nb repertoire of the library was then expressed in phages after rescue with the VCS helper phage. After three rounds of panning, 24 clones of the second round and 23 clones of the third round of panning were picked randomly for antigen-binding screening. The cell lysates of 28 clones scored positive in ELISA after detection with a mouse anti-HA antibody (Covance) and an alkaline phosphatase–anti-(mouse IgG) conjugate (Sigma). Sequence analysis revealed four unique sequences.

The corresponding plasmids containing DNA fragments encoding the GAK-specific Nbs were transformed into non-suppressor WK6 *E. coli* cells for recombinant protein expression in the periplasm. Cultures in TB (Terrific broth; 2.3 g of KH_2_PO_4_, 16.4 g of K_2_HPO_4_·3H_2_O, 12 g of tryptone, 24 g of yeast extract and 4 ml of 100% glycerol) medium supplemented with 0.1% glucose were induced with 1 mM IPTG overnight at 28°C. Cells were harvested by centrifugation (11300 ***g*** for 8 min at 4°C) and subjected to an osmotic shock to obtain the periplasmic extract. The recombinant proteins were purified by Ni-affinity and size-exclusion (Superdex S75) chromatography. The pure Nbs were stored at 4°C in 20 mM Tris/HCl (pH 8.0) and 125 mM NaCl.

### Kinetics of GAK–kinase inhibitor and GAK–Nb interaction

Interaction analyses of GAK with Nbs were performed by SPR (surface plasmon resonance) using a Biacore 3000 optical biosensor (GE Healthcare) at 25°C with a flow rate of 30 μl/min. All samples were diluted in analysis buffer composed of 50 mM Tris/HCl (pH 7.4), 150 mM NaCl, 50 μM EDTA and 0.005% Tween 20. Biotinylated GAK was immobilized on to the CAP chip surface using the Biotin CAPture kit (GE Healthcare) at capture levels between 60 and 120 RU (resonance units). Serial 2-fold dilutions of the respective Nbs were injected for 3 min. After recording the dissociation, analysis buffer supplemented with 1.5 M NaCl was injected for 1 min. No additional surface regeneration step was needed due to complete dissociation of Nbs.

Kinase inhibitor binding to GAK was characterized using a Biacore T100 instrument (GE Healthcare) at 25°C with a flow rate of 100 μl/min in HBS/DMSO analysis buffer [20 mM Hepes (pH 7.4), 150 mM NaCl, 50 μM EDTA, 0.005% surfactant P20 and 3% DMSO]. For each analysis cycle, GAK was freshly immobilized on to a CAP chip surface resulting in reproducible capture levels (between 720 and 3400 RU depending on the molecular mass of the respective inhibitor). The amount of GAK on a sensor chip surface was kept as low as possible in order to minimize secondary effects such as mass transport limitation and rebinding.

Serial 3-fold dilutions of kinase inhibitors were injected for 1 min with various dissociation times. Solvent correction was applied to all datasets.

Sensorgrams were processed and analysed using BIAevaluation software version 4.1.1 (for Biacore 3000) or Biacore T100 evaluation software version 2.0.3. Double referencing of all binding curves was applied as described in [18]. The binding data were fitted to a 1:1 Langmuir interaction model to calculate the rate constants for association (*k*_a_) and dissociation (*k*_d_) and the equilibrium dissociation constant (*K*_D_=*k*_d_/*k*_a_) for kinase inhibitor and Nb interactions.

### Crystallization of GAK

SeMet-labelled GAK was treated with 1 mg/ml chymotrypsin and incubated for 2 h on ice. However, chymotrypsin did not cleave GAK in solution. The integrity of the protein in the measured crystal was not tested and we cannot exclude that the catalytic domain or termini were cleaved during crystallization. Crystallization was performed in 400-nl sitting drops at 16°C using a reservoir solution containing 1.0 M succinic acid (pH 7.0) and 0.1 M Bis-Tris propane (pH 7.0). Viable apo crystals were cryoprotected in mother liquor supplemented with 25% glycerol before flash-freezing in liquid nitrogen.

### Complex purification and crystallization

Both GAK and Nb proteins were initially buffer-exchanged in 50 mM Hepes (pH 7.5) and 100 mM NaCl, and mixed at a 1:1.2 molar ratio. After 2 h of incubation on ice, the complexes were prepared by size-exclusion chromatography to remove the excess Nbs, and were then concentrated to 10–15 mg/ml. The GAK–NbGAK_1 and GAK–NbGAK_4 complexes were pre-incubated with 1 mM inhibitors, and crystallized using the sitting-drop vapour-diffusion method at 4°C or 20°C respectively. Crystals were obtained under various conditions (Supplementary Table S1 at http://www.biochemj.org/bj/459/bj4590059add.htm) and were cryoprotected in mother liquor supplemented with ethylene glycol before flash-freezing in liquid nitrogen.

### Data collection, structure solution and refinement

Two-wavelength peak (0.9791 Å) and inflection (0.9794 Å) MAD diffraction data for apo-GAK were collected at the 19-ID beamline of the Structural Biology Center at the Advanced Photon Source at Argonne National Laboratory using the program SBC collect and processed and scaled with HKL3000. The apo structure was solved by the two-wavelength MAD method with the SHELX programs from the HKL3000 suite using a Se anomalous signal, and the final model was refined using REFMAC and peak data. For the Nb–GAK complexes, diffraction data were collected at the Diamond Light Source (Harwell, U.K.), and processed either with MOSFLM [19] or XDS [20] before subsequent scaling with SCALA from the CCP4 suite [21]. The structures of the complexes were solved by molecular replacement with the PHASER program [22] using the co-ordinates of apo-GAK and the Camelid single-domain antibody NbHuL6 [23] as search models. Density modification was performed using the PARROT program [24], and the improved phases were used for automated model building in BUCCANEER [25]. The initial output structures were subjected to iterative cycles of manual model rebuilding using COOT [26] alternated with refinement in Buster [27] for the GAK–NbGAK_1 structure or REFMAC [28] for the GAK–NbGAK_4 structures. The complete models were checked for geometric correctness with MolProbity [29]. Data collection and refinement statistics are summarized in Supplementary Table S1.

### *In vitro* kinase assay

An ADP Glo™ assay (Promega) was carried out according to the manufacturer's instructions in buffer containing 50 mM Tris/HCl (pH 7.4) and 1 mM DTT. In brief, reactions were performed in a 25-μl volume using GAK and the histone H1 substrate (Calbiochem) at a concentration of 250 nM and 0.2 mg/ml respectively. The reactions were initiated by the addition of 100 μM ATP and 5 mM MgCl_2_, before incubation for 1 h at room temperature (22°C) with shaking. To measure the effect of Nbs or inhibitors on the activity of GAK, the reactions were supplemented with the Nbs or inhibitors at a 5-fold molar excess of the kinase. Data analysis was performed using GraphPad Prism.

### AUC

Sedimentation velocity experiments were performed at 4°C in a Beckman Optima XL-I Analytical Ultracentrifuge using a Ti-50 rotor at 98607 ***g***. The proteins used were in PBS buffer and at a concentration of ~20 μM. Data were analysed with SEDFIT [30] to the calculate c(*s*) distributions, which were then normalized into the sedimentation coefficients in water at 20°C (*s°_20,w_*) using the values for the solvent density (ρ_o_) of 1.0056 g/ml, viscosity (η_o_) of 1.0198×10^−2^ and the protein partial specific volume of 0.7309 ml/g.

### Inhibitor screening

Thermal stability-shift assays were carried out using a Real-Time PCR Mx3005p machine (Stratagene). In brief, GAK at 2 μM was mixed with 10 μM kinase inhibitors. The experiments and data evaluation for the melting temperatures were performed according to previously described protocols [31,32].

## RESULTS

### Structure of the apo-GAK kinase domain

**Figure 1:**
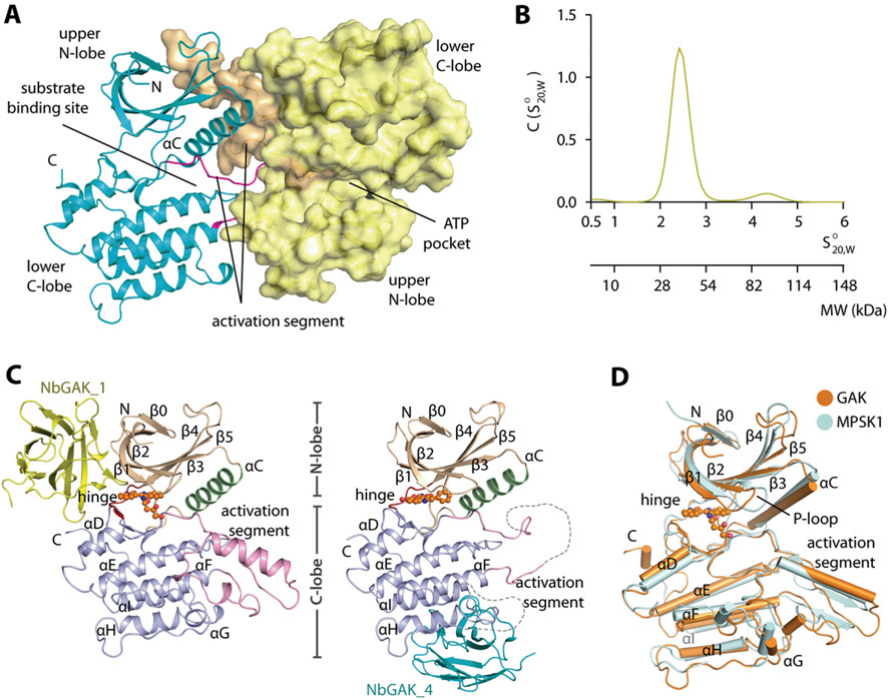
Overview of GAK and its Nb complexes (**A**) Dimeric assembly of GAK in the apo structure, which is enabled by an exchange in the extended activation segments. (**B**) Sedimentation velocity AUC of GAK demonstrates an existence of mainly monomeric kinase in solution with a possibility of a weak dimeric formation. Also see Supplementary Figure S1(B) (http://www.biochemj.org/bj/459/bj4590059add.htm). (**C**) Ribbon representations of the structures of GAK–NbGAK_1 (left-hand panel) and GAK–NbGAK_4 (right-hand panel), in which the Nbs are coloured yellow and cyan respectively. Secondary structure elements of the kinase are labelled and kinase inhibitors are shown as sphere. (**D**) Superimposition of GAK and MPSK1 revealing a highly conserved overall topology.

Crystallization of the GAK kinase domain (residues 14–351) yielded apo crystals that diffracted to 2.1 Å resolution. Overall, GAK adopts the typical bi-lobal kinase architecture with most of the structural elements characteristic of kinases in both the N- and C-lobes highly maintained. Two kinase molecules occupied the asymmetric unit of the hexagonal unit cell and associated in a head-to-toe arrangement (Figure 1A). In that arrangement, the largely disordered activation segment (Asp^181^–Ile^230^) dislodged from the lower kinase domain and bound to the upper kinase lobe of the interacting protomer. Using AUC we confirmed dimerization of GAK in solution, which was, however, weak and therefore not detected in the gel-filtration experiments (Figure 1B and Supplementary Figure S1 at http://www.biochemj.org/bj/459/bj4590059add.htm).

### Overview of the GAK–Nb complex structures

In order to study in more detail the activation mechanism of GAK we strived to generate additional crystal forms of the kinase using Nbs for stabilization. Peripheral blood lymphocytes from a GAK-immunized dromedary were isolated and served to clone the gene fragment repertoire of the antigen-binding domains present on the camel-specific heavy chain-only antibodies. Four Nbs with unique sequences were identified from a library (1.2×10^7^ individual transformants and 78% correctly sized inserts), which had been panned on recombinant GAK.

All four successfully isolated GAK-specific Nbs formed tight complexes with the GAK catalytic domain. Of these four, two Nbs, NbGAK_1 and NbGAK_4, yielded crystals. The asymmetric unit of the primitive monoclinic *P*2_1_ crystals of GAK–NbGAK_1 contained two molecules of the complex, whereas the GAK–NbGAK_4 complex crystals exhibited a *C-*centred monoclinic *C*2 lattice comprising only one complex in the asymmetric unit (Figure 1C). The differences in the crystal packing of these complexes were mainly due to the presence of the Nbs, which acted as additive binders to provide additional contact surfaces. This also led to the alteration of both interactions between kinase molecules within the crystals and, hence, the conformation of the kinase, which are explained in detail below. A summary of the statistics for data collection and refinement is shown in Supplementary Table S1.

The kinase domain of GAK was well defined in the electron density map of both structures. The GAK–NbGAK_4 complex showed a similar dimeric arrangement as observed in the apo structure with large portions of the activation segment and the αG helix (residues 265–274) disordered. In contrast, a near complete kinase molecule (residues 25–334) was seen in the GAK–NbGAK_1 structure revealing that the structure of the folded form of the activation segment was reminiscent of the unusual activation loop architecture described for MPSK1 (Figure 1D). Superimposition of the kinase from the two complexes revealed high similarity in the core kinase domain (Cα RMSD of 0.9 Å). The N-terminal region further formed an additional β-strand (β0) that joined and extended further the canonical N-lobe β-sheet. Similar to the observations for the unliganded GAK form, a structural comparison of GAK with MPSK1 demonstrated similarity in their overall kinase topology (Cα RMSD of ~2.0 Å) despite sharing only ~26% sequence identity (Figure 1D), suggesting conservation of the main structural elements within this highly diverse kinase family.

### Structural variations of Nbs

**Figure 2:**
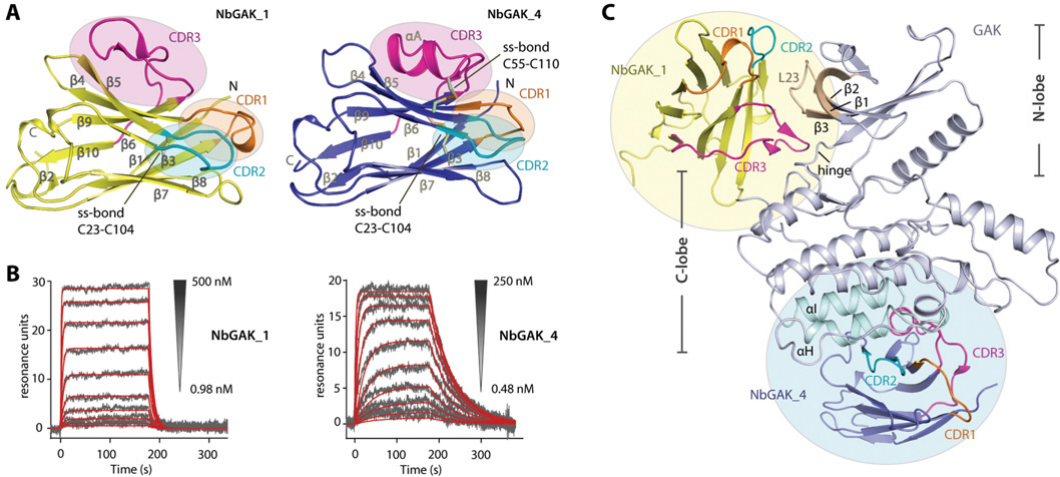
Analysis of specific NbGAK_1 and NbGAK_4 binding to GAK (**A**) Overall structures of the two Nbs demonstrate a classical β-sheet immunoglobulin scaffold. Their CDR regions are highlighted and disulfide bridges are indicated. For detailed structural and sequence analysis of the Nb interaction see Supplementary Figure S2 (http://www.biochemj.org/bj/459/bj4590059add.htm). (**B**) Interaction analysis of GAK with NbGAK_1 and NbGAK_4. Grey curves represent the measured responses, whereas red lines reflect the applied global fit using the 1:1 Langmuir interaction model. (**C**) Different epitopes of GAK are recognized by two Nbs. NbGAK_1 (yellow) binds to the N-terminal lobe, whereas NbGAK_4 (pale blue) recognizes the helical architectures of the C-terminal lobe of the kinase. For details see Supplementary Figures S3(A) and S3(B) (http://www.biochemj.org/bj/459/bj4590059add.htm).

Both NbGAK_1 and NbGAK_4 comprise ~120 amino acids, and their structures contained a single-domain immunoglobulin fold consisting mainly of β-strands from which two layers of β-sheets are formed (Figure 2A). These two layers of the sheets are linked by a conserved disulfide bond between framework regions 1 (FR1), and 3 (FR3), a characteristic that lends stability to the Nbs [11]. Although their core domains were structurally highly conserved (Cα RMSD of 1.2 Å), several structural differences were observed in all three of their CDRs (Figure 2A and Supplementary Figure S2 at http://www.biochemj.org/bj/459/bj4590059add.htm). Most notable is the third loop (CDR3 or H3) of NbGAK_4, which forms a unique helical segment created by a patch of hydrophobic amino acids (Figure 2A and Supplementary Figure S2). The overall differences in their CDRs play an important role regarding their antigen-binding properties and result in their different binding affinities for GAK. The SPR analysis revealed that NbGAK_1 binds to the kinase with a *K*_D_ value of 60 nM, a nearly 6-fold-lower affinity than that of NbGAK_4 (*K*_D_=10 nM) (Figure 2B and Table 1). Although no crystals could be obtained with NbGAK_2, this Nb showed the highest affinity to GAK target in comparison with all other Nbs tested in direct binding experiments using SPR (Table 1), whereas no rate and equilibrium-binding constants could be determined in a direct binding assay for the fourth Nb due to unspecific binding (results not shown).

**Table 1:** Kinetics of the interaction between NbGAK4 and NbGAK1 with GAK Calculated rate constants and equilibrium-binding constants for the binding of NbGAK_1 and NbGAK_4 to biotinylated GAK. Rate constants for all experiments were determined using a global fit analysis. *K*_D_ values represent the ratio of *k*_d_ to *k*_a_. A Langmuir 1:1 binding was employed to analyse the binding data in perfect agreement with the model, indicating direct interaction of GAK with each of the Nbs tested.

	Kinetic global fit					
Analyte	*k*_a_ (M^−1^·s^−1^)	*k*_d_ (s^−1^)	*K*_D_ (nM)	*R*_max_ (RU)	Percentage active	Steady-state analysis *K*_D_ (nM)
NbGAK_4	1.8×10^6^	1.8×10^−2^	10.3	19.8	67.7	12.8
NbGAK_1	2.2×10^6^	1.3×10^−1^	59.9	32.5	70.5	60.3
NbGAK_2	3.3×10^6^	9.7×10^−4^	0.294	22.1	78.6	–

### Nbs confer different epitope recognition sites

Superimposition of the two Nb complex structures revealed different recognition sites in GAK (Figure 2C). Although NbGAK_1 binds to the N-lobe, in particular β1, β2, β3, L23 loop and the hinge, the αH and αI helices are the regions in the C-lobe that are recognized by NbGAK_4. Although several regions of the kinase, including the activation segment and parts of the C-lobe, are disordered in the GAK–NbGAK_4 structure, the distant binding site of this Nb is unlikely to interfere with the folding of these structural elements (Figure 2C).

Both global shape and electrostatic complementarities between the Nb epitope-recognition sites and the GAK antigens provide a structural basis for selection of the binding site (details in Supplementary Figures S3A and S3C at http://www.biochemj.org/bj/459/bj4590059add.htm). NbGAK_1 makes specific contacts to the extended feature of the L23 loop through a shallow elongated cavity formed by the CDR regions. In addition the protruding CDR3 reaches underneath the N-lobe contacting the kinase β-sheet (numbering of Nbs based on the IMGT scheme from [33]). In contrast, the rather surface-filled CDRs of NbGAK_4 favour the shallower flat binding cavity created by helices αH and αI. In addition to the overall shape complementarity, the number of local intermolecular hydrogen bonds observed between the kinase antigens and the residues from the antigen-recognition sites provide a high degree of epitope discrimination and specificities (Supplementary Figures S3B and S3D).

### GAK dimerization

**Figure 3:**
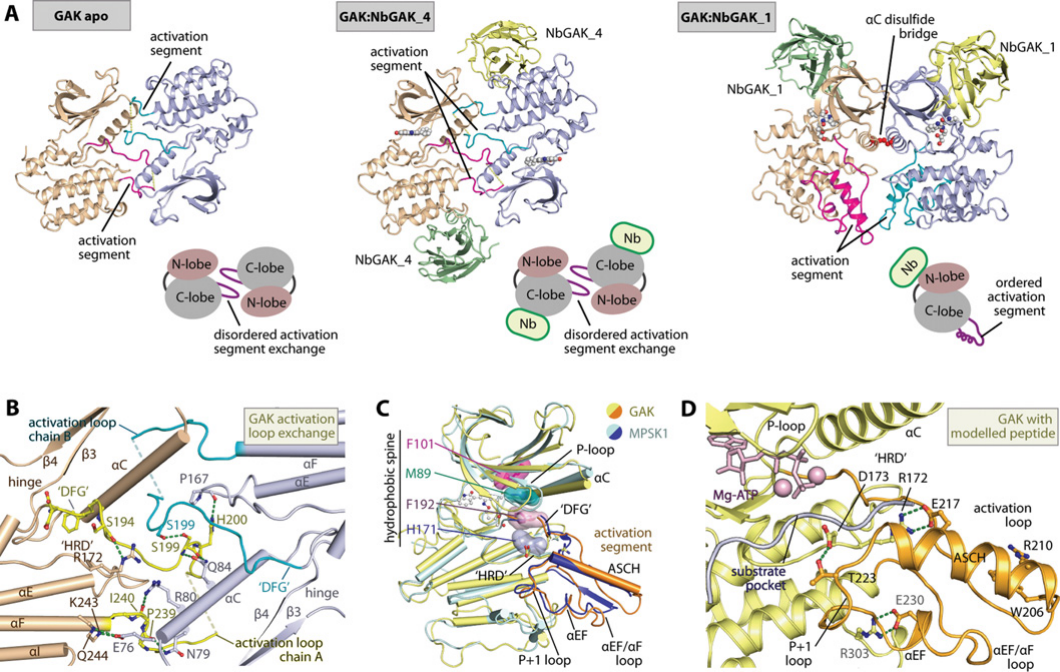
Nb_GAK1 and Nb_GAK4 capture different activation states of GAK (**A**) Arrangement of GAK oligomeric states in the crystals of dimeric apo-GAK (left-hand panel), dimeric GAK–NbGAK_4 (middle panel) and monomeric GAK–NbGAK_1 (right-hand), in which each kinase molecule is coloured wheat and pale blue, and their activation segments are in magenta and cyan. In the apo and NbGAK_4-complexed structures, formation of a homodimer in a head-to-tail fashion is assisted by an activation segment-exchange mechanism, whereas this is not observed in a fully ordered activation segment that prompts a monomeric form in the NbGAK_1-complexed crystals. The disulfide bridge formed between two cysteine residues from the αC observed in the GAK–NbGAK_1 complex is coloured red. (**B**) Details of intersubunit contacts within the activation segment-exchange region of the GAK–NbGAK_4 structure. (**C**) Superimposition of GAK from the NbGAK_1-complexed structure and MPSK1 reveal similar topology of the fully ordered activation segment with two highly conserved unique features, the ACSH and the parallel loop within the activation loop. This conformation of GAK also demonstrates a formation of the hydrophobic regulatory spine (R-spine), a characteristic of an active kinase. (**D**) Conformation of the activation segment confers an active state of GAK. Interactions between the HRD motif residues to both ASCH Glu^217^ and P+1 loop Thr^223^ residues, including communication between the αEF Glu^230^ to the C-lobe Arg^303^, lock the typically flexible activation segment in place, and this creates a platform for substrate binding. Modelling of the substrate peptide (grey ribbon) and ATP-MgCl_2_ (pink stick and ball) from the PKA structure (PDB code 1ATP) into GAK suggests a suitable pocket for accommodation of a substrate peptide for phosphorylation.

The dimeric assembly of GAK is a phenomenon not only observed in the apo form, but also in the crystals of the GAK–NbGAK_4 complex (Figure 3A). An application of the crystallographic two-fold symmetry to the complex subunit in the asymmetric unit results in a dimeric assembly of the kinase catalytic domain, which highly resembles that of the apo structure. The N-lobe αC and the C-lobe activation segment are the parts of the catalytic domain that are located at the two-fold interface and primarily mediate intermolecular contacts. In contrast, although containing two molecules of the complex in the asymmetric unit, the limited intermolecular contacts suggest that the GAK–NbGAK_1 complex depicts the kinase in its monomeric active form. In this monomer, no activation segment exchange between the two kinase molecules is observed, and the activation segments of both kinase molecules are fully ordered, a particularly interesting feature discussed below. However, a disulfide bridge formed between the two Cys^87^ located in the αC helices link the two molecules together in the GAK–NbGAK_1 complex. It is probable that this covalent linkage has been introduced during crystallization and is not relevant in the reducing environment of the cellular cytosol.

### Activation segment-mediated dimerization confers an inactive conformation

The complex of GAK with NbGAK_4 traps the catalytic domain in an inactive conformation, in which the activation segment exerts a domain-swap conformation. Although the P+1 loop and the αEF of the activation segment are disordered, the activation loop and the αEF/αF loop are aligned in a parallel manner and traverse the neighbouring molecule to be in proximity to the αC (Figure 3B). This leads to several peripheral intermolecular contacts in the dimeric self-assembly, including a network of hydrogen bonds. In addition, at the crossing point of two activation segments an exchange interaction between two Ser^199^ side chains and an intermolecular contact between His^200^ and the Pro^167^ main chain stabilize further the dimerization.

The engaged and partly unfolded activation segment in GAK in combination with the disordered αG leave the front of the active site with a large void that precludes the formation of a binding groove for substrates. The structural features in this complex therefore probably depict an inactive conformation of GAK on the basis of the dynamic properties of its activation segment.

### NbGAK_1 stabilizes an active conformation of the activation segment

In comparison with apo-GAK and the GAK–NbGAK_4 complex, significant structural rearrangements of the activation segment are observed in the GAK–NbGAK_1 complex. The structural changes are characterized by a major positional shift of the entire activation segment with a swing towards the C-lobe inducing an ordering of helix αG and the entire activation segment itself. The architecture of the GAK activation segment is similar to the one reported for MPSK1, as indicated by an RMSD of 1.7 Å in the superimposition of this region (Figure 3C). Stabilization of this conformation is achieved through various interactions, including hydrogen bonds between the main chain atoms of His^200^ from the N-terminal part of the activation loop to Pro^239^ and Ile^240^ located in the αE/αF loop. These contacts prompt a unique parallel loop section within the activation segment (Supplementary Figure S4 at http://www.biochemj.org/bj/459/bj4590059add.htm). The C-terminus of the activation loop thereby adopts a unique α-helix, a structural feature that has been predicted to be conserved within the NAK family [8]. The arginine and aromatic amino acid-rich content within the unique helix ASCH and its surrounding area enables the formation of several cation-π stacking interactions, for example between Trp^206^ and Arg^210^ and Arg^211^ and Tyr^235^ (Supplementary Figure S4).

An ordering of the activation segment is a hallmark of an active kinase, typically complemented by the closure and alignment of the N- and C-lobes and by the formation of the regulatory hydrophobic spine [34,35]. In the NbGAK_1 complex β4 Phe^101^, αC Met^89^, the DFG motif Phe^192^ and the HRD motif His^171^ align to form the regulatory spine (Figure 3C). Phe^101^, however, does not align efficiently with the other spine residues leading to a distortion. Furthermore, another important role of the ordered activation segment is to create a substrate-binding site and link the catalytic loop (HRD motif) with the activation segment. The latter is typically accomplished in kinases that require activation segment phosphorylation by polar interactions between phosphorylated activation loop residues and the HRD motif arginine residue. Interestingly, GAK mimics these interactions with a stretch of acidic residues in ASCH, in particular the central Glu^217^, which forms a salt bridge with the HRD motif Arg^172^ (Figure 3D) explaining why GAK is constitutively active and does not require phosphorylation of its activation segment. Further stabilization of this activation segment conformation is provided by a hydrogen bond between Thr^223^ at the tip of the P+1 loop and the HRD motif Asp^173^ and a conserved salt bridge between Glu^230^ from the αEF and the αH/αI Arg^303^. The latter provides a cross communication between the activation segment and the C-lobe in an active state [36]. Superimposition of the PKA–ATP–peptide complex (PDB code 1ATP) revealed that a docking groove created within the active site of GAK would be suitable to accommodate a peptide substrate (Figure 3D), which therefore suggests that this conformation of the activation segment may serve as an active conformation of the enzyme to enable peptide binding. However, the tight interaction of NbGAK_1 with the upper lobe induces structural changes that distort slightly the catalytic spine alignment and the upper lobe itself, which also affects the conserved salt bridge between Lys^69^ and αC Glu^85^ increasing the optimal distance of 3 Å seen in the GAK–NbGAK_4 structure to a distance of 4 Å (Supplementary Figure S5A at http://www.biochemj.org/bj/459/bj4590059add.htm).

**Figure 4:**
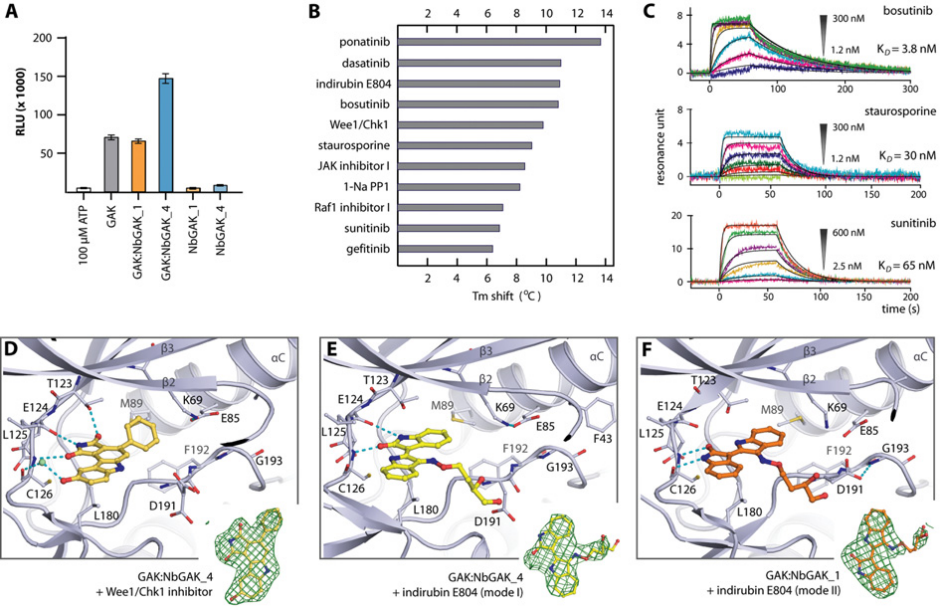
Kinase activities of GAK and its binding with inhibitors (**A**) Kinase activities of GAK in the absence or presence of the Nbs. Results are means±S.E.M. (**B**) Δ*T*_m_ values for several commercially available kinase inhibitors. (**C**) SPR binding assays of some inhibitors to GAK. Detailed interactions between Wee1/Chk1 (**D**) and indirubin E804 (**E** and **F**) and the kinase with the insets showing the |*F*_o_|−|*F*_c_| omitted map contoured at 3σ. Note that two modes of binding of indirubin E804 are observed in two different complexes.

To assess the effect of the bound Nbs on the activity of GAK, we performed *in vitro* kinase assays using histone H1 as a substrate [37] (Figure 4A). GAK was shown to be constitutively active in the absence of phosphorylation of the activation loop. Interestingly, its activity was slightly enhanced by the addition of NbGAK_4, but not by NbGAK_1, which is in contrast with the active conformation of the kinase trapped in the complex structure by NbGAK_1, but not NbGAK_4. NbGAK_2 and NbGAK_5 did not have any significant effect on the activity of GAK (results not shown). Structural analyses suggest that modest increase in enzymatic activity may be due to the more stable C-lobe in the GAK–NbGAK_4 complex as indicated by the generally lower *B*-factors in the NbGAK_4 complex (Supplementary Figure S5B). Potentially, the observed NbGAK_1 distortion of the upper N-lobe and the induced misalignment of the catalytic spine may offset any contributions to the stimulation of kinase activity that may result in stabilization of the folded activation segment in this monomeric state (Supplementary Figure S5C). In addition, due to the low concentrations used in enzyme kinetic assays any inhibiting effects by the very weak catalytic domain dimerization are expected to only minimally disturb catalytic activity.

### Inhibitor binding

To gain information on chemical scaffolds that could target GAK, we profiled the kinase against a collection of kinase inhibitors using thermal stability-shift assays, which identify inhibitor binding by a shift in the melting temperature (Δ*T*_m_) (Figure 4B) [38]. A number of diverse compounds showed strong temperature stabilization, suggesting potent binding of the screened inhibitors. Interaction of the clinically approved drugs ponatinib, dasatinib, bosutinib, sunitinib and gefitinib gave rise to Δ*T*_m_ value of 6.4–13.8°C, typically observed for inhibitors with a low nanomolar activity. Interestingly, the EGF1R inhibitor gefitinib, which is thought to cause some adverse effects due to its off-target activity towards GAK, showed the lowest Δ*T*_m_ value among the clinical inhibitors studied. Verification of some of the inhibitors by measuring the Δ*T*_m_ value using SPR confirmed that the binding affinities of these drugs were in the low nanomolar range. The trend in increasing affinity was in agreement with the higher Δ*T*_m_ value shifts, showing that bosutinib (Δ*T*_m_ of 10.8°C and *K_D_* of ~3.8 nM) was a better inhibitor of GAK than staurosporine (Δ*T*_m_ of 9.1°C and *K*_D_ of ~30 nM) and sunitinib (Δ*T*_m_ of 6.8°C and *K*_D_ of ~65 nM) (Figure 4C and Table 2).

**Table 2:** Kinetic constants and equilibrium dissociation constants of selected kinase inhibitors binding to GAK Results are means of at least two independent experiments.

	Kinetic global fit (1:1 interaction)			
Kinase inhibitor	*k*_a_ (M^−1^·s^−1^)	*k*_d_ (s^−1^)	*K*_D_ (nM)	Steady state *K*_D_ (nM)
Bosutinib	3.6×10^6^	1.4×10^−2^	3.8	
Staurosporine	2.4×10^6^	7.1×10^−2^	30	31.9
Sunitinib	7.5×10^5^	5.0×10^−2^	65.8	64.8

In order to provide insight into the binding modes of some of the identified inhibitors, several inhibitors were used in co-crystallization, but only two compounds, the Wee1/Chk1 kinase inhibitor and indirubin E804 which produced ~72% and ~90% GAK inhibition at 10 μM respectively, yielded crystals that enabled successful structure determination. All co-crystallized compounds were clearly defined by the electron density map and showed a typical ATP mimetic type-I binding mode (Figures 4D–4F). The Wee1/Chk1 inhibitor binds with its phenyl ring located deep towards the back pocket (Figure 4D). The planar carbazole core is placed along the kinase hinge where it forms hydrogen-bond networks between the pyrolidine-dione moiety to the Thr^123^ side chain, and the carbonyl and amine main chain atoms of Glu^124^ and Cys^126^ respectively. The hydroxy group at the other end of the carbazole core interacts with the Cys^126^ carbonyl atom via a water molecule. Interestingly, the indirubin E804 complex exhibits two binding modes which can be explained by the near-symmetrical core scaffold (Figures 4E and 4F). Two molecular contacts between the inhibitor and the kinase hinge residues are conserved in both orientations.

## DISCUSSION

Our previous work on MPSK1 revealed several molecular features predicted to be unique for the NAK family including an atypical activation segment [8]. The structural models shown in the present paper demonstrate that GAK shares similar activation segment architecture as described for MPSK1, suggesting conservation of this unique topology in NAKs. Networks of polar interactions link the activation segment with the catalytic loop HRD motif providing a structural rational for the constitutive activity of GAK.

Both the apo-GAK and GAK–NbGAK_4 complex crystallized as dimers with their disordered activation segments exchanged between the monomers. However, although intermolecular contacts in the GAK–NbGAK_4 dimer and in the GAK apo structure were primarily mediated by a large interface formed by the N-lobe αC and the C-lobe activation segment, the dimer observed in the GAK–NbGAK1 complex showed few intermolecular contacts. It is therefore probable that the observed dimer represents only a crystal contact region rather than an interface important for GAK activity. In addition, the putative dimer seen in the asymmetric unit of the GAK–NbGAK_1 complex was linked by a disulfide bridge formed between two cysteine residues located in helix αC. It is unlikely that a dimer is formed by an external/highly exposed disulfide bond in cells under the physiological normal reducing conditions present in the cellular cytosol. Therefore we consider each molecule to represent a monomeric state of the kinase with an active conformation of the enzyme that associated during crystallization due to oxidation of surface cysteine residues.

However, dimeric arrangements of catalytic domains with domain-exchanged activation segments have been described for a number of kinases including LOK (lymphocyte-oriented kinase), SLK (STE20-like kinase), CHEK (checkpoint kinase 1) and DAPK3 (death-associated protein kinase 3). These conformations facilitated *trans*-autophosphorylation at non-consensus sites in the activation segment [39]. In contrast with those dimeric kinase structures, the activation segment in dimeric GAK does not position a residue amenable for phosphorylation transfer into the active site of the interacting protomer. It is therefore not probable that the dimeric inactive state of GAK observed in GAK–NbGAK_4 as well as the apo structure is important for GAK autophosphorylation. This observation is in agreement with the constitutive activity of this kinase which does not require phosphorylation of the activation segment. The potential biological function of the observed dimerization in two structures, as well as in solution, therefore remains to be investigated. Dimerization of GAK *in vitro* has only been observed at a high protein concentration, which may, however, occur in cells. GAK seems to be located at the *trans*-Golgi and focal adhesions [40], probably contributing to the uncoating of clathrin vesicles, and the accumulation of GAK may occur at these sites leading to high local concentration and, as a consequence, to dimerization and inactivation of the kinase domain. In addition to the N-terminal kinase domain, GAK harbours other domains in its C-terminus, which are highly homologous with auxillin, namely an N-terminal tensin domain, a clathrin-binding domain and a J-domain. However, so far, none of these domains has been implicated in dimerization [2,40].

Nb-identification technology has been developed during the last two decades and recombinant Nbs have found wide applications in various diverse fields such as cancer diagnostics [41,42] anti-thrombotic therapy in coronary artery disease [43] and immunotherapy, or as a tool for use in cell biology such as developing specific binders for ChIP assays [44], protein purification [45] and crystallization [12,13]. Nbs possess a variety of features that make them ideally suited for crystallization studies. They are highly soluble due to specific hydrophilic amino acids found in the framework-two region between CDR1 and CDR2. They are very stable both in terms of temperature resistance as well as against denaturing effects and, therefore, are resistant to the chemicals used in crystallization trials, and they can be produced in large amounts in a variety of host systems including bacteria [11]. Nbs bind to their antigens with a high affinity, usually in the nanomolar range as confirmed in the present study. However, no direct correlation could be observed in the present study between affinity and the ability to co-crystallize. The high-affinity antibody Nb_GAK2, with a *K*_D_ value of 294 pM, did not result in co-crystals.

In the two GAK–Nb complexes two conformations of the activation segment have been observed, echoing the flexible nature of this region. The Nb co-crystal structure using NbGAK_1 displayed an ordered activation segment conformation and an active kinase conformation suggesting a high degree of intrinsic plasticity and a highly dynamic ability to switch between the inactive and active states through its flexible activation segment. Despite trapping of GAK in the active conformation NbGAK_1 had no influence on kinase activity, whereas binding of NbGAK_4 resulted in a small, but reproducible, increase in kinase activity. Thus both Nbs act primarily as binders that can provide crystal contacts compatible with either an active or an inactive conformation of the kinase domain. Thus using the generated Nbs in crystallization experiments provided a tool for sampling the conformational space of this dynamic protein kinase and for the reproducible crystallization of GAK in these two key conformations. This will be particularly important for the development of inhibitors that target either the active or the inactive state of GAK. The characterized binders also provide useful tools to study the role of GAK with traditional cell biology methods and can be used, for instance, in combination with a fluorescent tag to recognize endogenous GAK in cells.

Very few crystal structures of kinases in complex with specific binders have been solved so far [46,47]. Specific DARPins (designed ankyrin-repeat proteins) have been generated recognizing the phosphorylated active or unphosphorylated inactive form of ERK2 (extracellular-signal-regulated kinase 2), providing insight into the flexibility of the activation loop in the phosphorylated compared with the non-phosphorylated form [47]. Development of a specific DARPin facilitated the crystallization of the kinase structure PLK1 (polo-like kinase 1) [46]. More recently, co-crystal structures of EGFR have been solved with three different Nbs, of which two block the movement of EGFR and the third interferes with ligand binding due to an overlap in the binding site [48]. Nbs have also been used for crystallization of specific conformations of non-kinase proteins. The published structure of the active conformation of the β_2_ adrenoceptor in complex with an Nb [15] revealed, for the first time, the agonist-bound active state of the GPCR (G-protein-coupled receptor) helping to stabilize this transitional conformation of the receptor and providing insight into the structural rearrangements during β_2_ adrenoceptor activation. Thus Nbs are developing into versatile tools for stabilizing specific conformations of enzymes and therefore facilitating the crystallization and structural determination of diverse activation states. Owing to the high plasticity of the kinase catalytic domain these high-affinity binders are of particular interest for the elucidation of structural mechanisms regulating the catalytic activity of these dynamic molecules.

Respiratory side effects in the treatment of human carcinomas due to off-target inhibition have been described for the EGFR kinase inhibitor gefitinib (Iressa), limiting the therapeutic benefit of this drug [7]. These side effects have been linked to the inhibition of GAK as knockout mice expressing the kinase-dead form of GAK display similar respiratory dysfunctions. The present study confirms that not only does gefitinib potently inhibits GAK, but also other clinical kinase inhibitors, such as dasatinib a tyrosine kinase inhibitor approved for the treatment of CML (chronic myelogenous leukaemia) and Ph+ALL (Philadelphia chromosome-positive acute lymphoblastic leukaemia), as well as sunitinib another tyrosine kinase inhibitor approved for the treatment of RCC (renal cell carcinoma) and imatinib-resistant GIST (gastrointestinal stromal tumour), inhibit GAK even more potently. Respiratory side effects observed in patients treated with dasatinib or sunitinib may be associated with inhibition of GAK [49], although a direct link to GAK has not been established so far. The availability of the GAK crystal structure provides the basis for the development of more specific inhibitors for the treatment of these cancers.

## Online data

Supplementary data
