# Supplementary material for: Structure of cyclin G-associated kinase (GAK) trapped in different conformations using nanobodies
Source: Biochem J. 2014 Mar 14;459(Pt 1):59–69. doi: 10.1042/BJ20131399 (PMC3957475; doi:10.1042/BJ20131399)
Supplement: Supplementary data [file bj4590059add.pdf]

Biochem. J. (2014) **459**, 59–69 (Printed in Great Britain) doi:10.1042/BJ20131399

SUPPLEMENTARY ONLINE DATA

Structure of cyclin G-associated kinase (GAK) trapped in different conformations using nanobodies

Apirat CHAIKUAD*[1], Tracy KEATES*[1], Cécile VINCKE†, Melanie KAUFHOLZ‡, Michael ZENN§, Bastian ZIMMERMANN§, Carlos GUTIÉRREZ¶, Rong-guang ZHANG||, Catherine HATZOS-SKINTGES||, Andrzej JOACHIMIAK||, Serge MUYLDERMANS†, Friedrich W. HERBERG‡§, Stefan KNAPP* and Susanne MÜLLER*[2]

*University of Oxford, Target Discovery Institute (TDI) and Structural Genomics Consortium (SGC), Old Road Campus Research Building, Oxford OX3 7DQ, U.K.
†Research Unit of Cellular and Molecular Immunology and Department of Structural Biology, VIB, Vrije Universiteit Brussel, Pleinlaan 2, 1050 Brussels, Belgium
‡Department of Biochemistry, University of Kassel, Heinrich-Plett Strasse 40, 34132 Kassel, Germany
§Biaffin GmbH & CoKG, Heinrich-Plett Strasse 40, 34132 Kassel, Germany
¶Department of Animal Medicine and Surgery, Veterinary Faculty, University of Las Palmas de Gran Canaria, 35416, Arucas, Las Palmas, Spain
||Midwest Center for Structural Genomics and Structural Biology Center, Biosciences Division, Argonne National Laboratory, 9700 South Cass Avenue, Argonne, IL 60439, U.S.A.

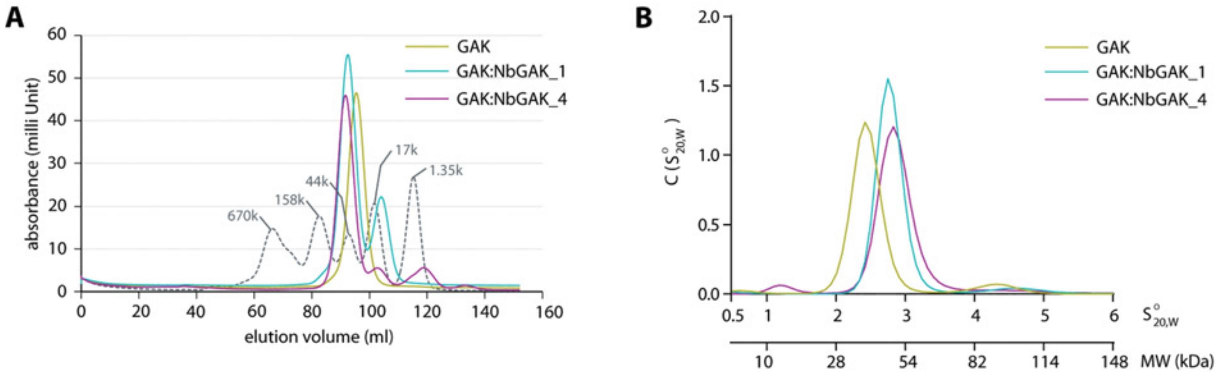

Figure S1 Monomeric GAK in solution

(**A**) Size-exclusion chromatography profiles of the GAK and Nb complexes performed on Superdex S200 column. (**B**) Sedimentation velocity AUC of the GAK and Nb complexes performed at 98 607 ***g*** using a protein concentration of ~20 μM. The peak at 1.2 S corresponds to the free Nb; the kinase domain has a sedimentation coefficient of 2.5S and the kinase Nb complexes sediment around 3S. Some dimerization is obvious at 4.5S.

[1] These authors contributed equally to the work.
[2] To whom correspondence should be addressed (email susanne.muller-knapp@sgc.ox.ac.uk).
The crystal structures reported in this paper have been deposited in the PDB under codes 4O38, 4C57, 4C58 and 4C59.

© 2014 The Author(s)
The author(s) has paid for this article to be freely available under the terms of the Creative Commons Attribution Licence (CC-BY) (http://creativecommons.org/licenses/by/3.0/) which permits unrestricted use, distribution and reproduction in any medium, provided the original work is properly cited.

Biochemical Journal www.biochemj.org

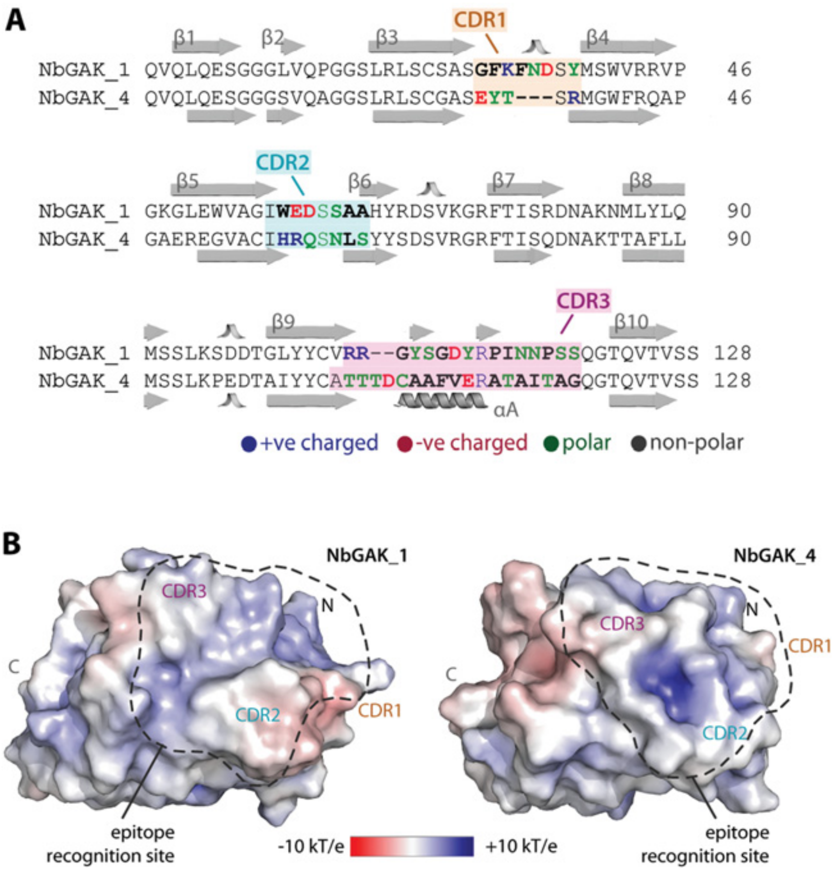

Figure S2 Details of structural comparison of NbGAK_1 and NbGAK_4

(**A**) Structure-based sequence alignment with their corresponding secondary structures annotated. High amino acid variations are observed for the three variable CDRs, and are coloured according to charge properties. NbGAK_4 has a shorter than normal CDR1 (H1). Highest sequence variation is found in CDR3 (H3), where a stretch of hydrophobic amino acids results in a helical segment (αA) for NbGAK_4 compared with a short anti-parallel two-stranded β-sheet insertion for NbGAK_1. (**B**) Surface representation and electrostatic potentials demonstrate distinct architectures of the epitope-recognition sites between the two Nbs. The GAK-binding site of NbGAK_4 is defined by a surface-filled non-charged interaction site with positively charged pockets spreading along the CDR surface. In contrast the epitope-binding site of NbGAK_1 forms a shallow groove between the CDR3 on one side and CDR1 and CDR2 on the other side, and exhibits a broad positively charged electrostatic surface. Calculation of solvent-accessible area using the PISA server revealed a slightly larger contact surface on GAK (692 Å^2) for the NbGAK_1 interaction, compared with the 578 Å^2 surface area needed for tight complex formation with NbGAK_4.

© 2014 The Author(s)
The author(s) has paid for this article to be freely available under the terms of the Creative Commons Attribution Licence (CC-BY) (http://creativecommons.org/licenses/by/3.0/) which permits unrestricted use, distribution and reproduction in any medium, provided the original work is properly cited.

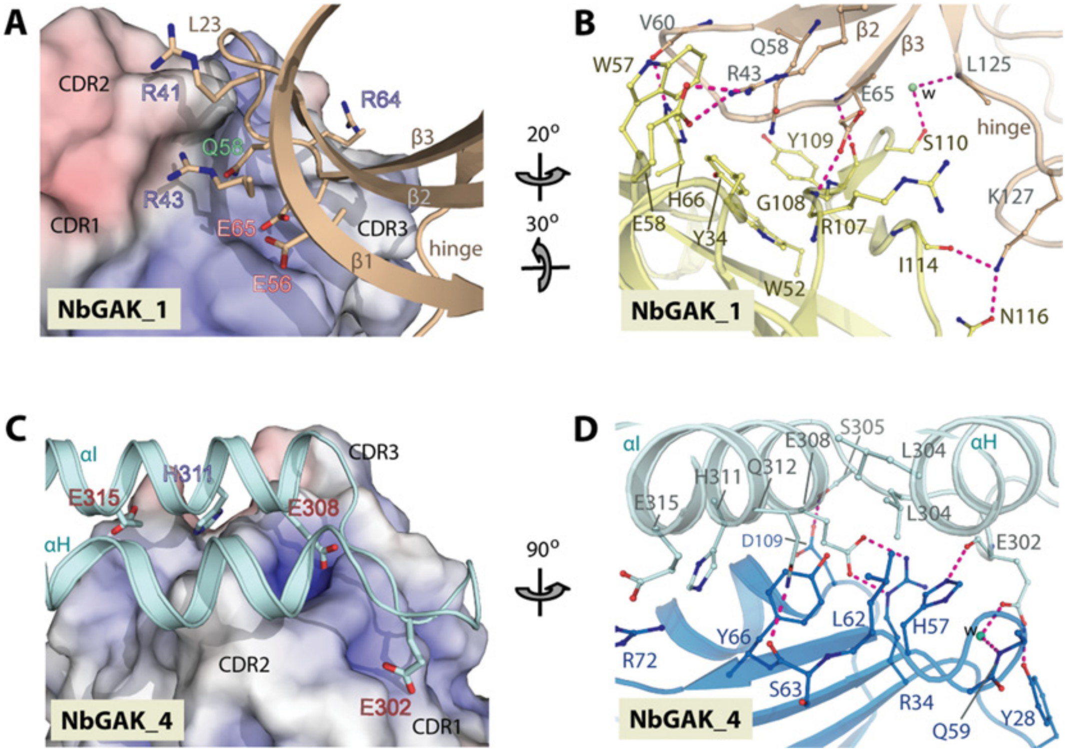

Figure S3 Intermolecular interaction between GAK and Nbs

Surface representation of the Nbs reveals shape and electrostatic complementarities between their epitopes and CDRs, which distinguish the binding sites of NbGAK_1 (**A**) and NbGAK_4 (**C**) on to the kinase. Local intermolecular contacts including hydrogen bonds (distance <3.2 Å) and salt bridges between the kinase and NbGAK_1 (**B**) and NbGAK_4 (**D**) are represented by broken lines. Some interactions are mediated by water molecules, which are shown as green spheres.

© 2014 The Author(s)
The author(s) has paid for this article to be freely available under the terms of the Creative Commons Attribution Licence (CC-BY) (http://creativecommons.org/licenses/by/3.0/) which permits unrestricted use, distribution and reproduction in any medium, provided the original work is properly cited.

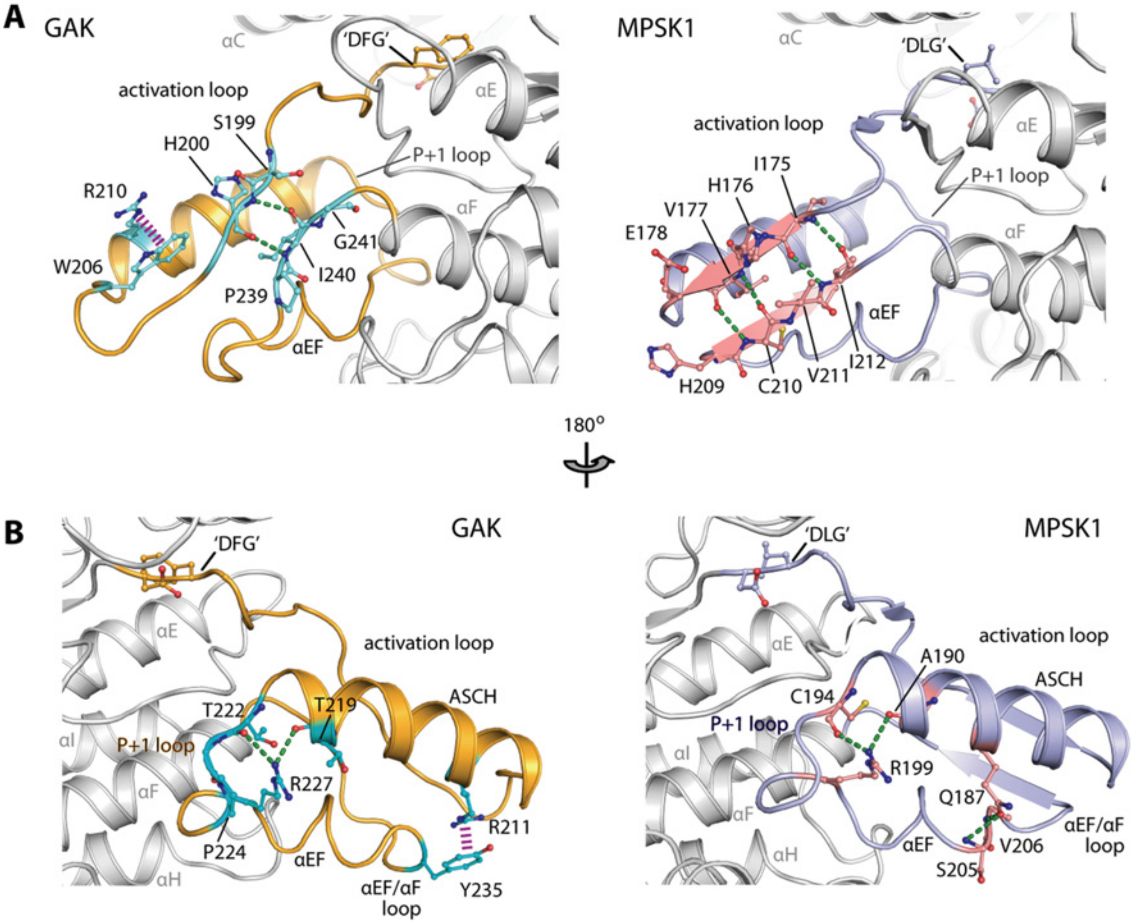

Figure S4 Stabilizing interactions of the active conformation of the activation segment in GAK

Comparison of detailed interactions between residues of GAK (left-hand panel) and MPSK1 (right-hand panel) within the activation loop (**A**) and the ASCH segment (**B**) of the active conformation of the activation segments.

© 2014 The Author(s)
The author(s) has paid for this article to be freely available under the terms of the Creative Commons Attribution Licence (CC-BY) (http://creativecommons.org/licenses/by/3.0/) which permits unrestricted use, distribution and reproduction in any medium, provided the original work is properly cited.

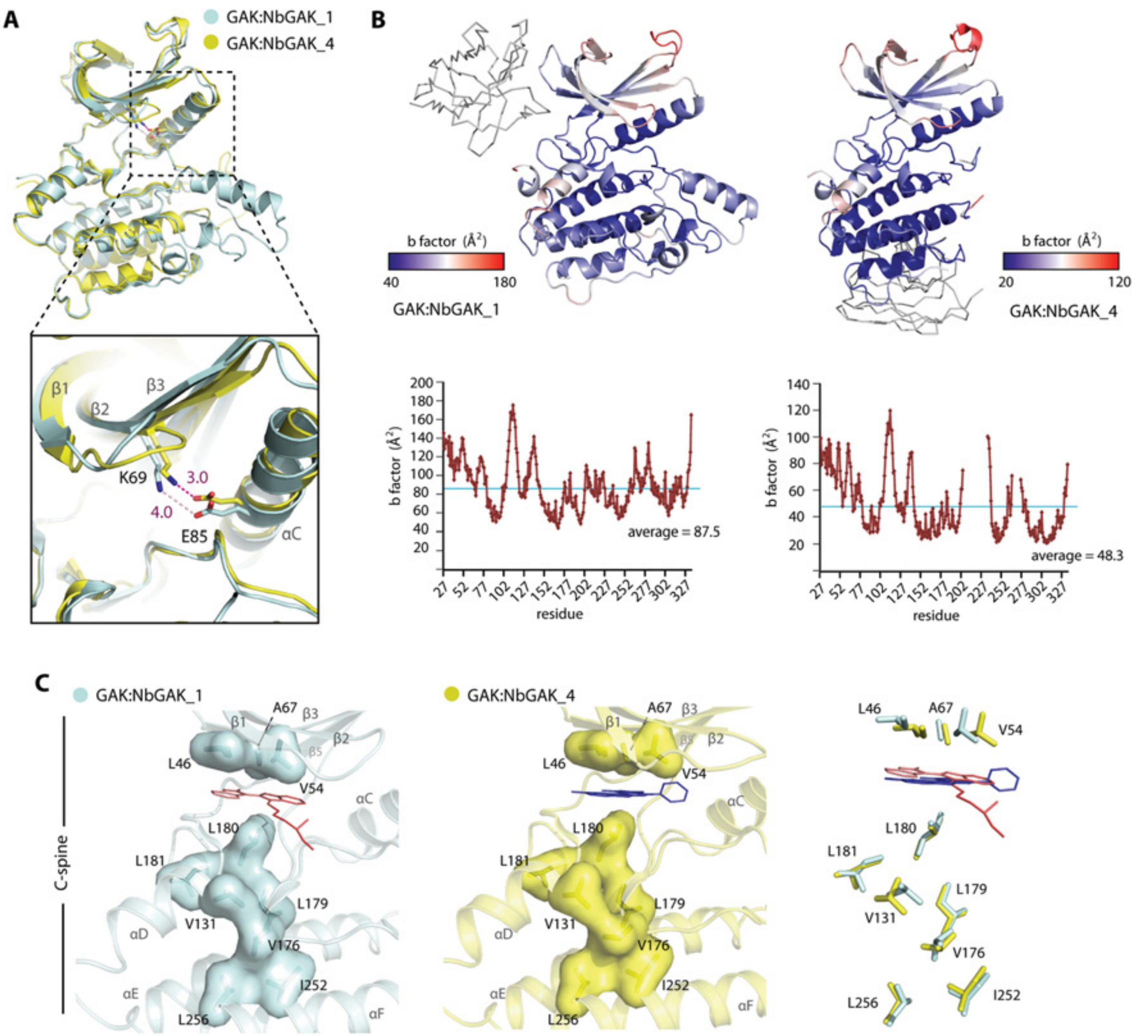

Figure S5 NbGAK_1 distorted the GAK N-lobe and its overall stability

(**A**) Superimposition between GAK molecules from two Nb complexes reveals a distorted relatively open N-lobe in the GAK–NbGAK_1 complex that restricts salt bridge formation. (**B**) Cartoon representations with colour mapping of temperature (*B*-) factors on the kinase. In addition to the N-lobe, the C-lobe of GAK in the NbGAK_1-complexed structure showed a higher degree of flexibility compared with that of the kinase in the GAK–NbGAK_4 complex. (**C**) Comparison of the residue compositions of the C-spine in both complexes. Slight shifts in the positions of some residues are observed between the two structures (right-hand panel).

© 2014 The Author(s)
The author(s) has paid for this article to be freely available under the terms of the Creative Commons Attribution Licence (CC-BY) (http://creativecommons.org/licenses/by/3.0/) which permits unrestricted use, distribution and reproduction in any medium, provided the original work is properly cited.

Table S1 Data collection and refinement statistics

Values in parentheses are for the highest-resolution shells. P/L/O, protein, ligand molecules and other (water and solvent molecules).

Complex	Apo–GAK		GAK–NbGAK_1 indirubin E804	GAK–NbGAK_4 Wee1/Chk1	GAK–NbGAK_4 indirubin E804
PDB code	4O38		4C57	4C58	4C59
Data collection	Inflection	Peak			
Beamline	APS 19-ID	APS 19-ID	Diamond, I02	Diamond, I04	Diamond, I04
Wavelength (Å)	0.9794	0.9791	0.9795	1.0121	1.0121
Resolution (Å)	50–2.22 (2.26–2.22)	74.11–2.10 (2.14–2.10)	83.39–2.55 (2.69–2.55)	45.09–2.16 (2.28–2.16)	19.75–2.80 (2.95–2.80)
Space group	$P3_221$	$P3_221$	$P2_1$	$C2$	$C2$
Cell dimensions					
a, b, c (Å)	103.6, 103.6, 132.1	103.4, 103.4, 132.0	75.2, 86.7, 83.9	$a = 156.9$, $b = 37.4$, $c = 76.0$ Å	$a = 174.3$, $b = 37.8$, $c = 75.6$ Å
α, β, γ (°)	90.0, 90.0, 120.0	90.0, 90.0, 120.0	90.0, 96.4, 90.0	90.0, 108.5, 90.0	90.0, 111.5, 90.0
Number of unique reflections	41031 (1,999)	48311 (2356)	34717 (4842)	22748 (3292)	11580 (1678)
Completeness (%)	99.9 (100)	99.9 (100.0)	99.1 (95.1)	99.2 (99.7)	99.5 (99.8)
$I/\sigma I$	41.3 (4.1)	36.6 (2.2)	10.5 (2.1)	10.3 (2.0)	8.3 (2.0)
R_{merge} (%)	7.3 (75.7)	8.2 (95.7)	9.1 (64.1)	10.9 (77.7)	14.1 (69.1)
Redundancy	14.5 (14.7)	14.5 (14.0)	5.2 (3.5)	5.7 (5.7)	3.6 (3.7)
Phasing	MAD	MAD	Molecular replacement	Molecular replacement	Molecular replacement
Resolution (Å)	74.11–2.1	74.11–2.1			
Phasing power	1.32	1.32			
Figure of merit					
Acentric/centric	0.84/0.8	0.84/0.8			
After density modification	0.92	0.92			
Refinement					
Number of atoms in refinement (P/L/O)		4314/–/282	6676/54/223	3020/25/206	2976/27/63
R_{fact} (%)		16.5	17.8	17.9	20.2
R_{free} (%)		19.5	22.8	24.2	26.2
B_f (P/L/O) (Å^2)		57/–/56	80/78/62	44/34/45	61/70/41
RMSD bond (Å)		0.016	0.010	0.014	0.008
RMSD angle (°)		1.6	1.1	1.6	1.0
MolProbity					
Ramachandran favour (%)		96.6	96.7	96.1	97.6
Ramachandran allowed (%)		100.0	100.0	100.0	100.0
Crystallization	1.0 M succinic acid (pH 7.0) and 0.1 M Bis-Tris propane (pH 7.0)		14% PEG 3350, 0.2 M sodium sulfate, 0.1 M Bis-Tris propane (pH 7.0) and 10% ethylene glycol	12% PEG 3350 and 0.13 M magnesium formate	9% PEG 3350 and 0.06 M magnesium formate

Received 22 October 2013/16 January 2014; accepted 17 January 2014
Published as BJ Immediate Publication 17 January 2014, doi:10.1042/BJ20131399

© 2014 The Author(s)
The author(s) has paid for this article to be freely available under the terms of the Creative Commons Attribution Licence (CC-BY) (http://creativecommons.org/licenses/by/3.0/) which permits unrestricted use, distribution and reproduction in any medium, provided the original work is properly cited.
